# Suppression of Poly(rC)-Binding Protein 4 (PCBP4) reduced cisplatin resistance in human maxillary cancer cells

**DOI:** 10.1038/srep12360

**Published:** 2015-07-21

**Authors:** Yumi Ito, Norihiko Narita, Nozomi Nomi, Chizuru Sugimoto, Tetsuji Takabayashi, Takechiyo Yamada, Kazuhiro Karaya, Hideki Matsumoto, Shigeharu Fujieda

**Affiliations:** 1 Department of Otorhinolaryngology Head and Neck Surgery, Faculty of Medical Sciences, University of Fukui, 23 Shimoaizuki, Matsuoka, Eiheiji, Fukui, 910-1193, Japan; 2 Department of Otorhinolaryngology, Faculty of Medical Sciences, University of Oita; 3 Division of Bioresearch, Life Science Research Laboratory, Faculty of Medical Sciences, University of Fukui, 23 Shimoaizuki, Matsuoka, Eiheiji, Fukui, 910-1193, Japan; 4 Division of Oncology, Biomedical Imaging Research Center, Faculty of Medical Sciences, University of Fukui, 23 Shimoaizuki, Matsuoka, Eiheiji, Fukui, 910-1193, Japan

## Abstract

Cisplatin plays an important role in the therapy for human head and neck cancers. However, cancer cells develop cisplatin resistance, leading to difficulty in treatment and poor prognosis. To analyze cisplatin-resistant mechanisms, a cisplatin-resistant cell line, IMC-3CR, was established from the IMC-3 human maxillary cancer cell line. Flow cytometry revealed that, compared with IMC-3 cells, cisplatin more dominantly induced cell cycle G2/M arrest rather than apoptosis in IMC-3CR cells. That fact suggests that IMC-3CR cells avoid cisplatin-induced apoptosis through induction of G2/M arrest, which allows cancer cells to repair damaged DNA and survive. In the present study, we specifically examined Poly(rC)-Binding Protein 4 (PCBP4), which reportedly induces G2/M arrest. Results showed that suppression of PCBP4 by RNAi reduced cisplatin-induced G2/M arrest and enhanced apoptosis in IMC-3CR cells, resulting in the reduction of cisplatin resistance. In contrast, overexpression of PCBP4 in IMC-3 cells induced G2/M arrest after cisplatin treatment and enhanced cisplatin resistance. We revealed that PCBP4 combined with Cdc25A and suppressed the expression of Cdc25A, resulting in G2/M arrest. PCBP4 plays important roles in the induction of cisplatin resistance in human maxillary cancers. PCBP4 is a novel molecular target for the therapy of head and neck cancers, especially cisplatin-resistant cancers.

Cisplatin is used widely as a standard chemotherapeutic agent for therapy of human head and neck squamous-cell carcinoma (HNSCC). Recently, postoperative cisplatin-based chemo-radiotherapy has been accepted in many countries. It reportedly improves local and regional control, leading to improvement of disease-free survival of HNSCC^1^,^2^. However, regrowth or recurrence of cancer can occur after chemo-radio therapy, suggesting that resistance of HNSCC to cisplatin can be acquired^3^.

For some types of cancer, reports have described several mechanisms of cisplatin resistance. Glutathione mediates inactivation of cisplatin or export of cisplatin through the putative ATP-dependent glutathione S-conjugate export pump in ovarian cancer cells^4^. Cisplatin resistance results from induction of XRCC3 in breast cancer cells^5^. A previous report described that AKT1 induced cisplatin resistance in lung cancer cells through a mammalian target of the rapamycin (mTOR) signaling pathway^6^. Although cisplatin resistance is often observed in HNSCC, the underlying mechanisms of resistance remain unclear.

Poly(rC)-Binding Protein 4 (PCBP4) is an RNA-binding protein (RBP). It plays an important role in stabilizing mRNA and translation^7^,^8^. The PCBP family consists of hnRNP K, PCBP1, PCBP2, PCBP3 and PCBP4^9^. All PCBPs have hnRNP K homology (KH) domains with poly(C)-binding specificity and bind various DNA or RNA^9^. The PCBP4 gene located at 3q21 encodes at least four variants including MCG10, MCG10a, αCP-4, and αCP-4a^7^. MCG10 consists of 424 amino acid including two KH domains, three proline-rich domains, one potential nuclear export signal, and one potential nuclear localization signal^10^. MCG10a, an alternative splice variant of MCG10, is 55 amino acids shorter than MCG10 in the first KH domain^10^. MCG10 is a NH_2_-terminus truncated form of αCP-4 which has an additional KH domain^11^. Alpha CP-4a is an alternative splice variant of αCP-4 that has a shorter COOH-terminus without proline-rich domains, a nuclear export signal, and a nuclear import signal^7^.

The relation between PCBP family and various human cancers has been increasingly reported. The loss of PCBP1 in hepatic tumors can engender a metastatic phenotype^12^. An earlier report described that PCBP2 was downregulated considerably in oral cancers and that PCBP2 overexpression induced apoptosis^13^. In contrast, knockdown of PCBP2 inhibited glioma growth through the induction of apoptosis^14^. Reportedly, highly proliferative lung cancers have a lack of αCP-4 expression^15^. Moreover, induction of αCP-4 has been reported to induce cell cycle arrest in G2/M and to suppress tumor growth^11^. Overexpression of MCG10 or MCG10a induced cell cycle arrest in G2/M and apoptosis in human non-small cell lung carcinoma cells^10^. Therefore, we focused on PCBP family, especially PCBP4 in the present study, because PCBP4 is related to the G2/M cell cycle arrest and we analyzed G2/M arrest in HNSCC previously^16^.

To analyze unknown mechanisms of cisplatin resistance in HNSCC, we established the highly cisplatin-resistant IMC-3CR cell line from the human maxillary cancer IMC-3 cell line. Regarding cisplatin resistance, the IC_50_ of cisplatin were, respectively, 8.4 and 41.0 (μg/ml) in IMC-3 and IMC-3CR cells^17^. For this study, we specifically examined cell cycle arrest in G2/M induced by cisplatin in the HNSCC cell line. Cell cycle arrest in G2/M can allow cisplatin-damaged DNA to be repaired, leading to escape from apoptosis and to survival of cancer cells. Therefore, release of cisplatin-induced G/2M arrest can present a strategy to overcome cisplatin resistance. Through PCR array and flow cytometry analyses, the authors found that cisplatin treatment induced PCBP4 expression, induced more G2/M arrest, and induced less apoptosis in IMC-3CR cells compared to IMC-3 cells. Knockdown of PCBP4 by RNAi reduced cisplatin-induced G2/M arrest, resulting in enhanced apoptosis. MTT assay showed that suppression of PCBP4 by RNAi down-regulated cisplatin resistance in IMC-3CR cells. Consistent with this, overexpression of PCBP4 in IMC-3 cells showed enhanced cisplatin resistance. Additionally, we showed that PCBP4 can bind to Cdc25A and that it can reduce both mRNA and protein level of Cdc25A after cisplatin treatment, leading to G2/M arrest.

The results of our current study demonstrated that PCBP4 plays an important role in cisplatin resistance in HNSCC cells. PCBP4 is therefore a promising candidate to overcome cisplatin resistance. It might serve as part of a novel strategy of chemo–radiotherapy for HNSCC.

## Results

### Certification of PCBP4 variants expressed in IMC-3CR cells

Because PCBP4 gene reportedly encodes four transcripts including αCP-4, αCP-4a, MCG10, and MCG10a, we determined the major variants expressed in IMC-3CR cells using RT-PCR with the primers 335F/885R and 769F/1222R, with reference to an earlier report^7^. Only the expression of αCP-4 was observed (Supplementary Fig. S1), which is compatible with that earlier report^7^. Because αCP-4 is a major transcript of PCBP4 gene, we treat PCBP4 and αCP-4 equally hereinafter in this report.

### Cell cycle population after cisplatin treatment

**Figure 1 f1:**
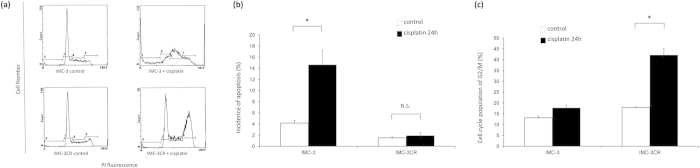
The cell cycle population was analyzed in IMC-3 and IMC-3CR cells using flow cytometry after cisplatin treatment (1 μg/ml, 24 h). (**a**) Original data of flow cytometry. Upper and lower panels respectively present results of IMC-3 and IMC-3CR cells. (**b**) Apoptosis (sub-G1) and (**c**) G2/M distributions are shown in bars. *P < 0.05. Error bars ± s.d.

The cell cycle population of IMC-3 and IMC-3CR cells was analyzed using flow cytometry after cisplatin treatment (1 μg/ml). Figure 1a presents the results of flow cytometry. The cell cycle populations of sub-G1 and G2/M were compared between IMC-3 and IMC-3CR cells. Figure 1b,[Fig f1]c respectively show the sub-G1 equivalent to apoptosis and G2/M population induced by cisplatin treatment for 24 h. In contrast to IMC-3 cells, sub-G1 was not induced in IMC-3CR cells by cisplatin (Fig. 1b). Cell cycle arrest in G2/M was observed to a more significant degree in IMC-3CR cells than in IMC-3 cells (Fig. 1c). These results suggest that IMC-3CR cells avoid apoptosis and that they survive through G2/M arrest, repairing DNA damaged by cisplatin.

### Screening of genes by PCR array

A PCR array system was used to investigate changes of the mRNA after cisplatin treatment in IMC-3CR cells. Analyses using PCR array revealed that the expression of several kinds of mRNA was altered by cisplatin treatment (1 μg/ml) for 6 h in IMC-3CR cells. In this study, we specifically investigated the genes for which cisplatin increased expression (Table 1), and especially genes related to G2/M cell cycle arrest^16^. The genes for which the ratio was higher than 2.0 were selected. We selected PCBP4 as the candidate of the target gene to examine resistance to cisplatin because PCBP4 reportedly induces cell cycle arrest in G2/M in some lung cancer cells^11^.

### Expression of PCBP4 in IMC-3CR cells

**Figure 2:**
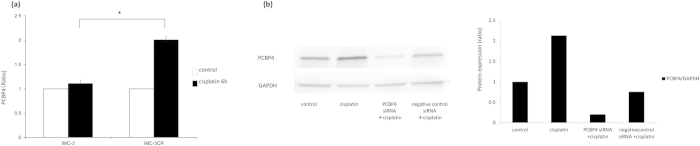
The mRNA and protein expression of PCBP4 were analyzed respectively using quantitative real-time PCR and Western blotting. (**a**) Quantitative real-time PCR. IMC-3 and IMC-3CR cells were treated with cisplatin (1 μg/ml) for 6 h. (**b**) Western blotting. IMC-3CR cells were treated with cisplatin (1 μg/ml) for 48 h. The quantified density of the blots is shown in the right panel. *P < 0.05. Error bars ± s.d. GAPDH is used as a loading control.

The mRNA expression of PCBP4 was validated using quantitative real-time PCR. Cisplatin treatment (1 μg/ml) for 6 h induced PCBP4 expression significantly in IMC-3CR cells, but not in IMC-3 cells (Fig. 2a). Next, protein expression of PCBP4 was analyzed by Western blotting in IMC-3CR cells. The protein of PCBP4 was enhanced by cisplatin treatment (1 μg/ml) for 48 h (Fig. 2b). To analyze the function of PCBP4, we transfected small interfering RNA (siRNA) for PCBP4 in IMC-3CR cells. Cisplatin-induced PCBP4 was suppressed completely by specific siRNA for PCBP4 and negative control siRNA did not affect it (Fig. 2b). Since PCBP4 can be induced by activated p53 in response to DNA damage^7^,^10^, the activation of p53 in IMC-3CR cells was analyzed after cisplatin treatment by Western blotting of phosphorylation at the Ser 15 residue, a hallmark of activated p53^18^ (Supplementary Fig. S2). The Western blot analysis showed that cisplatin induced phosphorylated p53 at least 6 h after treatment in IMC-3CR cells. All original scans of Western blot analysis can be found on-line as Supplementary Fig. S3.

### Functions of PCBP4 on cell cycle and cell viability

**Figure 3:**
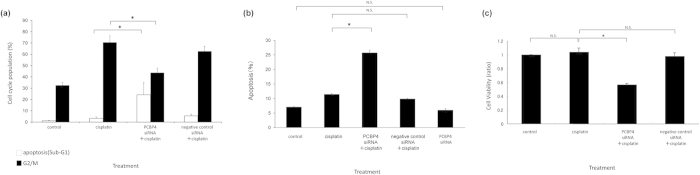
Functions of PCBP4 cells were analyzed using specific siRNA in IMC-3CR. (**a**) The cell cycle distribution was analyzed using flow cytometry after cisplatin treatment (1 μg/ml, 48 h). *P < 0.01. Error bars ± s.d. (**b**) Apoptotic cells were confirmed using Annexin V after cisplatin treatment (1 μg/ml, 48 h). *P < 0.05. Error bars ± s.d. (**c**) Cell viability was analyzed using MTT assay after cisplatin treatment (1 μg/ml, 48 h). *P < 0.05. Error bars ± s.d.

The functions of PCBP4 on the cell cycle under cisplatin stimulation were analyzed using flow cytometry in IMC-3CR cells. Similarly to the results presented in Fig. 1, cisplatin treatment (1 μg/ml) for 48 h induced cell cycle arrest in G2/M but not sub-G1 equivalent to apoptosis in IMC-3CR cells (Fig. 3a). Suppression of PCBP4 by RNAi decreased cisplatin-induced G2/M cell cycle arrest (71–44%) and enhanced sub-G1 (3–24%) significantly (Fig. 3a). Negative control siRNA did not affect the cell cycle population after cisplatin treatment. Apoptotic cells were detected using flow cytometry with Annexin V labeling (Fig. 3b). Results confirmed that suppression of PCBP4 by RNAi enhanced cisplatin-induced apoptosis (11.4–25.7%) significantly in IMC-3CR cells (Fig. 3b). The siRNA for PCBP4 alone did not affect the induction of apoptosis (Fig. 3b). It was confirmed that the siRNA for PCBP4 did not have cytocidal effects by MTT assay (Supplementary Fig. S4).

Next, we analyzed the effect of PCBP4 on cell viability by MTT assay (Fig. 3c). The cell viability was not decreased by cisplatin treatment (1 μg/ml, 48 h) in IMC-3CR cells (Fig. 3c). Suppression of PCBP4 by RNAi reduced the cell viability of IMC-3CR cells after cisplatin treatment (1 μg/ml, 48 h) by 57% (Fig. 3c), which is consistent with the induction of apoptosis in flow cytometric analysis (Fig. 3b). These results suggest that the suppression of PCBP4 impaired the cisplatin resistance of IMC-3CR cells through reduction of G2/M cell cycle arrest.

### Validation of function using overexpressing vector of PCBP4

**Figure 4:**
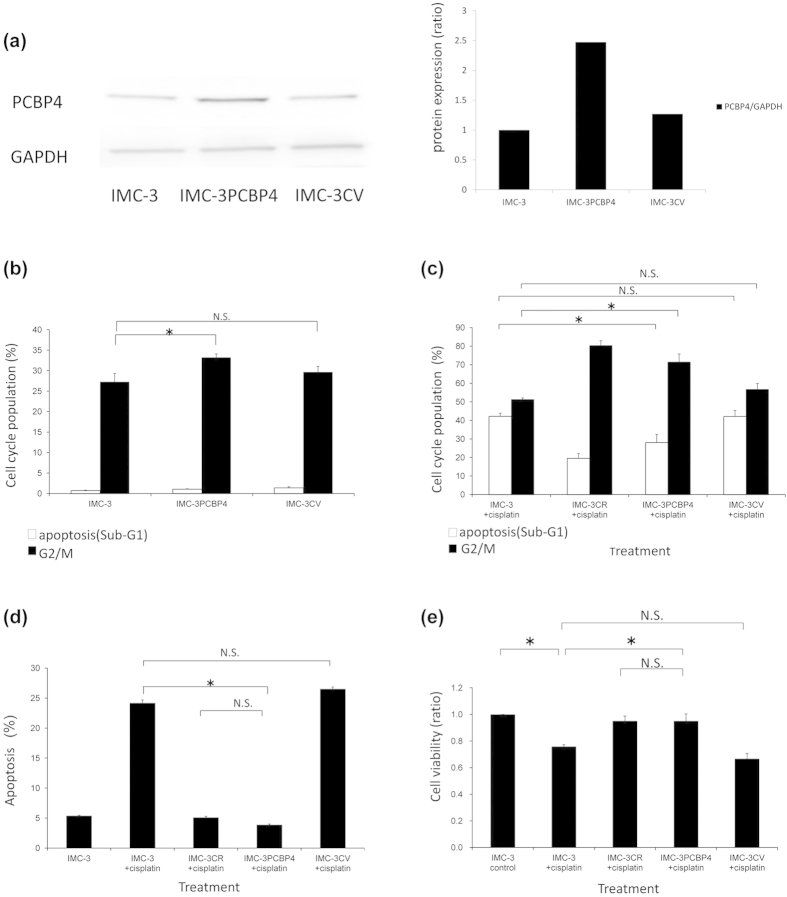
(**a**) Overexpression of PCBP4 protein in IMC-3PCBP4 cells w**a**s confirmed by Western blotting. GAPDH was used as a loading control. The quantified density of the blots is presented in the right panel. (**b**) The cell cycle population was observed using flow cytometry without stimulation. *P < 0.05. Error bars ± s.d. (**c**) The cell cycle population was analyzed using flow cytometry after cisplatin treatment (1 μg/ml, 48 h). *P < 0.05. Error bars ± s.d. (**d**) Apoptotic cells were confirmed using Annexin V after cisplatin treatment (1 μg/ml, 48 h). *P < 0.01. Error bars ± s.d. (**e**) Cell viability analyzed by MTT assay after cisplatin treatment (1 μg/ml, 48 h). *P < 0.05. Error bars ± s.d.

To validate and investigate the detailed functions of PCBP4, we generated IMC-3 cell line in which PCBP4 is expressed strongly through stable transfection of the PCBP4 overexpressing vector. The expression of PCBP4 was confirmed by Western blotting. The cell line with enhanced expression of PCBP4 were named IMC-3PCBP4 (Fig. 4a). The cell line transfected with an empty vector was named IMC-3CV for vehicle control. These cell lines were used to analyze the details of PCBP4 function.

Through analyses with flow cytometry, IMC-3PCBP4 showed more increased G2/M arrest than either IMC-3 and IMC-3CV with no stimulation 72 h later (IMC-3PCBP4 33.2% vs. IMC-3 27.2%, and IMC-3CV 29.7%, Fig. 4b). Nevertheless, the enhancement of G2/M arrest was less than had been expected, which implies that PCBP4 is not an inducer of G2/M cell cycle arrest but that it might sustain G2/M cell cycle arrest induced by cisplatin treatment.

Next, the cell cycle population was analyzed after cisplatin treatment (1 μg/ml, 48 h). As shown in Fig. 4c, IMC-3PCBP4 showed less sub-G1 equivalent to apoptosis and more G2/M cell cycle arrest than either IMC-3 or IMC-3CV (sub-G1: IMC-3PCBP4 28.0% vs. IMC-3 42.2%, and IMC-3CV 42.1%, G2/M: IMC-3PCBP4 71.5% vs. IMC-3 51.2%, and IMC-3CV 56.7%). The cell cycle population of IMC-3PCBP4 after cisplatin treatment was similar to that of IMC-3CR (sub-G1: 19.5%, G2/M: 80.3%).

Apoptotic cells were confirmed using flow cytometry with Annexin V labeling (Fig. 4d). Overexpressed PCBP4 in IMC-3PCBP4 reduced cisplatin-induced apoptosis significantly compared to IMC-3 or IMC-3CV cells (IMC-3PCBP4 3.9% vs. IMC-3 24.2%, and IMC-3CV 26.5%, Fig. 4d). No significant difference was found for cisplatin-induced apoptosis between IMC-3CR and IMC-3PCBP4 (IMC-3CR 5.1% vs. IMC-3PCBP4 3.9%).

The cell viability after cisplatin treatment was analyzed using MTT assay (Fig. 4e). IMC-3PCBP4 showed higher cell viability than either IMC-3 or IMC-3CV after cisplatin treatment (1 μg/ml, 48 h) (IMC-3PCBP4 95.1% vs. IMC-3 75.8%, and IMC-3CV 66.6%, Fig. 4e), suggesting that overexpression of PCBP4 brought IMC-3 cell cisplatin resistance equivalent to IMC-3CR.

### Analyses of regulator in cell cycle

**Figure 5:**
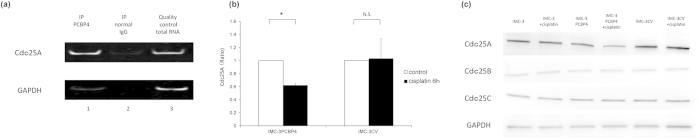
Cell cycle regulators in the downstream of PCBP4 were analyzed. (**a**) RNA immunoprecipitation and RT**-**PCR. Quality control total RNA means whole RNA before immunoprecipitation (lane 3). IP signifies immunoprecipitation by anti-PCBP4 specific antibody (lane 1) and normal IgG as a negative control (lane 2). GAPDH is shown as a housekeeping gene. (**b**) Expression of Cdc25A mRNA was analyzed using quantitative real-time PCR after cisplatin treatment (1 μg/ml, 6 h). *P < 0.05. Error bars ± s.d. (**c**) Protein levels of Cdc25A, Cdc25B, and Cdc25C were analyzed by Western blot after cisplatin treatment (1 μg/ml, 24 h). GAPDH is used as a loading control.

Because members of the PCBP family can mediate expression of other genes through combination with DNA or RNA, we expected that there should be a regulator of the cell cycle downstream of PCBP4. To investigate unknown targets of PCBP4, we specifically examined Cdc25A because Cdc25A reportedly engenders an accelerated G2/M phase transition through assembly and activation of CDK1-Cyclin B complexes^19^. RNA immunoprecipitation and RT-PCR were performed on IMC-3PCBP4 after cisplatin treatment (1 μg/ml, 24 h) to analyze the target mRNA of PCBP4. The results demonstrated that PCBP4 combined with mRNA of Cdc25A (Fig. 5a). Cdc25A was not detected from the immunocomplex of normal IgG as the control (Fig. 5a). PCBP4 did not combine with mRNA of Cdc25B or Cdc25C (data not shown).

Next, we investigated whether PCBP4 can affect the expression of Cdc25A or not. Real-time PCR demonstrated overexpression of PCBP4 reduced the mRNA level of Cdc25A by cisplatin treatment (1 μg/ml, 6 h) (Fig. 5b). Similarly, Western blot analyses showed that overexpression of PCBP4 in IMC-3PCBP4 reduced the protein level of Cdc25A after cisplatin treatment (1 μg/ml, 24 h) (Fig. 5c). In IMC-3 and IMC-3CV, cisplatin did not influence Cdc25A expression. The protein expression of neither Cdc25B nor Cdc25C was reduced by cisplatin in IMC-3PCBP4 cells (Fig. 5c). These results suggest that PCBP4 induces G2/M arrest through combination with Cdc25A and suppression of Cdc25A, resulting in cisplatin resistance.

## Discussion

DNA-damaging agents such as ionizing irradiation or chemotherapeutic drugs are known to induce G2/M cell cycle arrest^20^. Cancer cells can avoid apoptosis and survive through DNA repair during cell cycle arrest. Previously, some molecules were reported to release G2/M cell cycle arrest and to enhance apoptosis by DNA-damaging agents^21^. This phenomenon, called G2-checkpoint abrogation, is being investigated as a modality to enhance the efficacy of irradiation and chemotherapeutic drugs. Additionally, many cancer cells are known to include defective regulation of the G1 checkpoint, resulting in greater dependence on the G2 checkpoint than on normal cells^22^,^23^. Therefore, the G2-checkpoint abrogator can be a strategy for cancer-specific medicine.

The G2-checkpoint abrogators have been investigated for 20 years. Previous reports described that caffeine induced overriding G2 checkpoint and radiosensitization in G1-defective cancer cells^24^,^25^. Reportedly, Chk 1/2 inhibitor AZD7762 sensitized pancreatic cancer cells to radiation^26^. Although various agents targeting Chk 1/2, ATM, or ATR have been reported as G2-checkpoint abrogators^21^,^27^, PCBP families have not been reported.

This study revealed that cisplatin induced PCBP4 expression in cisplatin-resistant IMC-3CR cells and that suppression of cisplatin-induced PCBP4 reduced cisplatin resistance. Results also show that overexpression of PCBP4 engenders G2/M cell cycle arrest and cisplatin resistance. These results suggest that suppression of PCBP4 can enhance the effect of cisplatin through G2-checkpoint abrogation.

As for the mechanism of G2/M arrest by PCBP4, many underlying molecules are expected to be involved. Reportedly, p21 is a major inhibitor of G1/S cell cycle transition^28^. An earlier report described that PCBP4 can bind to 3′-UTR of p21 mRNA and reduce its stability, resulting in suppression of G1/S cell cycle arrest and facilitating G2/M arrest and/or apoptosis after cellular stress^7^. However, other aspects related to how PCBP4 induces G2/M cell cycle arrest directly have remained unclear. Referring to previous reports, overexpression of Cdc25A and Cdc25B, but not Cdc25C facilitated earlier assembly and activation of Cdk1-cyclin B complex, which is necessary for mitotic entry^19^. Similarly, repression of Cdc25A or Cdc25B by RNAi reportedly delays the G2/M transition, although repression of Cdc25C does not affect it^29^,^30^. The present study showed that PCBP4 can combine with Cdc25A and reduce its expression, inducing G2/M cell cycle arrest after cisplatin treatment. We demonstrated that overexpression of PCBP4 reduced only Cdc25A expression after cisplatin treatment, but it did not influence the level of Cdc25B and Cdc25C, which is compatible with reports suggesting that Cdc25A and Cdc25B can individually induce G2/M accumulation and delay of mitotic entry^29^. Furthermore, our results related to Cdc25A complement those of a previous report describing that PCBP4 is the inducer of G2/M cell cycle arrest through an unknown factor^7^. In addition, our results showed that p53 was activated after cisplatin treatment in IMC-3CR cells (Supplementary Fig. S2). Results suggest that *p53* status of IMC-3CR cells is wild and that p53 is activated by cisplatin, leading to the induction of PCBP4, the suppression of downstream Cdc25A, and G2/M cell cycle arrest.

Although we showed the overexpression of PCBP4 in IMC-3 cells induced G2/M cell cycle population, the induction was not dramatic, which is a similar result to that reported earlier^11^. The observation with cisplatin showed that overexpression of PCBP4 in IMC-3PCBP4 cells induced more G2/M cell cycle arrest than the control IMC-3 cells. These results suggest that PCBP4 by itself is not an inducer of G2/M cell cycle arrest, but a sustainer of G2/M cell cycle arrest after DNA damage such as cisplatin. Therefore, PCBP4 can be related to resistance to cisplatin. The suppression of PCBP4 engenders enhancement of chemosensitivity.

Previous reports have suggested that chemoresistance can be derived from various mechanisms in human malignancy^4^,^5^,^6^,^31^. ATP-binding cassette (ABC) transporters have been implicated in the resistance for platinum chemotherapeutic drugs^32^. ABC transporters, especially ABCC2, can mediate the active efflux of platinum chemotherapeutic agent across the cell membrane, leading to chemoresistance and poor prognosis in human cancers^32^. It is suggested that IMC-3CR cells possibly reduce the intracellular accumulation of cisplatin through overexpression of ABC transporters. In spite of the possibility of ABC transporters, our results suggested that PCBP4 played a major role in cisplatin resistance because the overexpression of PCBP4 increased the cisplatin resistance of IMC-3 cells almost to the same degree as IMC-3CR cells (Fig. 4c,d).

A previous report described the possibility of overexpressed PCBP4 as a tumor suppressor inducing G2/M arrest^11^. In contrast, our cells with overexpression of PCBP4 showed no remarkable increase of apoptosis or G2/M arrest (Fig. 4b). This discrepancy can be derived from the difference of cell properties and methods of experiments. Overexpression of some molecules is known not always to mimic their real functions, which means that PCBP4 within normal cellular amounts can induce G2/M arrest after DNA-damaging stimulation. Furthermore, PCBP4 evoked over the usual cellular level can engender growth delay or apoptosis. Then PCBP4 can be a G2-checkpoint abrogator in the response against cisplatin.

Although cisplatin is a standard chemotherapeutic agent for advanced HNSCC accompanied with radiation, its add-on effect is limited^1^,^2^. The development of chemosensitizers is necessary for strategies including cisplatin^3^. In our study, inhibition of PCBP4 decreased cisplatin resistance in head and neck cancer cells, which suggests that PCBP4 is a major component of cisplatin resistance and a new target for treatment of HNSCC, by enhancement of the effects of chemo-radiotherapy with cisplatin. Regimens based on inhibitors that are selective for PCBP4 might be particularly promising in cases for which therapeutic treatments for advanced head and neck cancers beyond surgery have had limited success to date.

## Methods

### Cell line, culture conditions, and drugs

Human maxillary squamous cell carcinoma IMC-3 cells (kindly provided by Dr. S. Komiyama, Kyushu University, Fukuoka, Japan)^33^ were maintained in conditioned medium [prepared from RPMI 1640 (Nissui Pharmaceutical Co. Ltd., Tokyo, Japan) supplemented with 2 mM L-glutamine, 100 units/ml penicillin, 100 μg/ml streptomycin, and 10% heat-inactivated FCS (Life Technologies Corporation, Carlsbad, CA, USA) ] at 37 °C under a humidified atmosphere of 5% CO_2_ in air.

Cisplatin was purchased from Bristol-Myers Squibb Co. (New York, NY, USA). The cisplatin-resistant cell line (IMC-3CR) was developed in the presence of increasing concentrations of cisplatin with repeated subcultures until the cells became fully resistant to cisplatin and could grow exponentially in the presence of 0.5 μg/ml of the drug for 2 weeks prior to being returned to drug-free medium. The drug-resistant cell lines were passed in drug free medium, and there was no loss of resistance during 6 months of experimentation^17^.

### Flow cytometric analysis of cell cycle population

We analyzed the cell cycle distribution of IMC-3 cells and IMC-3CR cells after cisplatin treatment using a flow cytometer (BD FACSCanto^TM^ II; Becton, Dickinson and Company, Franklin Lakes, NJ, USA) as described previously^16^. Similarly, the cell cycle distribution of IMC-3PCBP4 cells, in which PCBP4 is overexpressed, was analyzed. The sizes of sub-G1 and G2M fractions were calculated using BD FACSDiva^TM^ software. Furthermore, analysis of apoptotic cells was performed by the same systems, using an Annexin V-FITC Kit System for the Detection of Apoptosis (Beckman Coulter Inc., Brea, CA, USA) according to the manufacturer’s instructions.

### RNA extraction and reverse transcription

Total RNA was isolated using an RNeasy Mini kit (Qiagen, Hilden, Germany). Then the cDNA for quantitative real-time PCR was synthesized using a High-capacity cDNA Reverse Transcription Kit (Life Technologies Corporation) according to the manufacturer’s instructions.

### PCR Array

The relative mRNA expressions after cisplatin stimulation in IMC-3CR were analyzed using an RT^2^ Profiler^TM^ PCR array (Human DNA Damage Signaling Pathway, Qiagen) according to the manufacturer’s protocol^34^. Briefly, IMC-3CR cells (5 × 10^5^) were placed in RPMI 1640 supplemented with 10% heat-inactivated FCS. Cells with or without cisplatin (1 μg/ml) were incubated at 37 °C in a humidified atmosphere containing 5% CO_2_ for 6 h. After incubation, cells were washed with PBS, total RNA was extracted using RNeasy Mini Kit (Qiagen), and cDNA was synthesized through RT performed with an RT^2^ First Strand Kit (Qiagen) according to the manufacturer’s protocol. The cDNA was applied to the PCR array and real-time PCR performed on a Sequence Detection System (ABI PRISM^®^ 7000; Life Technologies Corporation) using PCR master mix (SA Biosciences RT^2^ qPCR Master Mix; Qiagen) for SYBR Green detection for each reaction. Samples were amplified with a precycling hold at 95 °C for 10 min, followed by 40 cycles of denaturation at 95 °C for 15 s and annealing for 1 min at 60 °C. The PCR array results were uploaded to the RT^2^ Profiler PCR Array Data Analysis website (http://pcrdataanalysis.sabiosciences.com/pcr/arrayanalysis.php). The alteration of mRNA expression was analyzed using ΔΔCt method.

### Quantitative real-time PCR

Quantification of mRNA levels of the target gene was performed using real-time fluorescence detection TaqMan technology and StepOnePlus^TM^ real time PCR system (Life Technologies Corporation) with Taqman^®^ Universal PCR Master Mix (Life Technologies Corporation). Primers used for these analyses were Taqman Gene Expression Assays (Life Technologies Corporation), including Hs99999905-m1 for GAPDH, Hs00263475-31 for PCBP4, Hs00947994-m1 for Cdc25A, Hs01550934-m1 for Cdc25B, and Hs00156411-m1 for Cdc25C.

### Western blot analysis

Cells were harvested and examined using Western blot analysis as described previously^16^. Cells were washed twice with ice-cold PBS and dissolved in solubilizing buffer (pH 7.4, 1% Triton X-100, 1% deoxycholic acid sodium salt, 0.1% SDS, 20 mM Tris-HCl, 150 mM NaCl, 5 mM EDTA, 1 mM PMSF, 10 mg/ml pepstatin, and 10 mg/ml leupeptin). Each aliquot of protein (50 μg) was used for Western blot analysis. After electrophoresis on 12.5% poly-acrylamide gels, the protein was transblotted to Hybond-P (GE Healthcare Life Sciences, Uppsala, Sweden) in transfer buffer (192 mM glycine, 25 mM Tris, 2.5 mM SDS, and 10% methanol). The blots were blocked with 3% nonfat dry milk in pH 7.4 TBST. Then they were incubated with anti-PCBP4 antibody (1:200), anti-Cdc25A antibody (1:100), anti-Cdc25B antibody (1:100), anti-Cdc25C antibody (1:200), anti-p53 (1:200), or anti-GAPDH antibody (1:400). These primary antibodies were purchased from Santa Cruz Biotechnology, Inc. (Dallas, TX, USA). The anti-phosphorylated p53 antibody was purchased from Cell Signaling Technology, Inc. (Danvers, MA, USA) and served for Western blotting (1:500). The blots were incubated secondarily with horse radish peroxidase-conjugated (HRP-conjugated) anti-rabbit or anti-mouse IgG antibody (Dako, Agilent Technologies Inc., Santa Clara, CA, USA). Subsequently, the blots were developed with chemiluminescence Western blot detection reagents (Dako) according to the manufacturer’s instructions. The blot density was quantified using Image Quant TL software (GE Healthcare Life Sciences).

### Analysis of cell viability by MTT assay

After cisplatin treatment, cell viability of IMC-3, IMC-3CR, IMC-3PCBP4, and IMC-3CV was determined by MTT (3-[4,5-dimethylthiazol-2-yl]-2,5 -diphenyltetrazolium bromide) assay referring to the previous report^35^. Briefly, MTT (0.4 mg/ml; Sigma-Aldrich, St Louis, MO, USA) was added to culture wells after washing with PBS. The converted dye was dissolved with dimethyl sulfoxide 2 h after incubation at 37 °C. The absorbance was measured at wavelengths of 540 nm and 630 nm using a microplate reader (Spectra Max 250; Molecular Devices, Sunnyvale, CA, USA).

### RNA interference

An siRNA for PCBP4 and a nonspecific negative control siRNA were purchased from Life Technologies Corporation (Stealth RNAi^TM^ siRNA). IMC-3CR cells were seeded to a 96-well plate (1 × 10^4^ cells/well) for MTT assay or 6-well plate (1 × 10^5^ cells/well) for flow cytometry without antibiotics. The siRNA was transfected with Lipofectamine^®^ 2000 Transfection Reagent (Life Technologies Corporation.) according to the manufacturer’s instructions. The suppression of PCBP4 protein was confirmed using Western blot analysis at 48 h after transfection. Each assay using siRNA was performed at least three times. The mean values were used for analyses.

### Plasmids

Total RNA of adult human liver was purchased from BioChain Institute Inc. (Newark, CA, USA). Reverse transcription was performed using a PrimeScript^®^ first strand cDNA synthesis kit (Takara Bio Inc., Otsu, Shiga, Japan) according to the manufacturer’s protocol. A cDNA fragment encoding amino acids 1–306 of PCBP4 was amplified by PCR using primers 355F (5′-ACA CAC TCG CAG GTC GCT GT-3′) and 1704R (5′-GCA GTG ATG AGG TAG AGG TAC TGG GC-3′). These NH_2_-terinal PCBP4 fragments were then cloned into the HindIII-EcoRI site of the pcDNA3.1 (+), a neomycin-regulated expression vector (Life Technologies Corporation).

### Ribonucleoprotein (RNP) Immunoprecipitation Assay (RIP Assay)

To analyze RNAs binding to PCBP4 protein, RIP Assay was performed using a RiboCluster Profiler^TM^ RIP Assay kit (Medical & Biological Laboratories Co., Ltd., Nagoya City, Aichi, Japan) following the protocols of the manufacturer. Briefly, IMC-3PCBP4 cells (1 × 10^7^) were treated with cisplatin (1 μg/ml) for 24 h. Lysis of the cells was performed after washing. The protein G beads (Pierce^TM^ Protein G Plus Agarose, Thermo Fisher Scientific, Waltham, MA, USA) were pre-incubated with anti-PCBP4 antibody (Santa Cruz Biotechnology Inc.) or normal IgG (Medical & Biological Laboratories Co., Ltd.). Then RNA-protein immunocomplexes were precipitated using the pre-incubated protein G beads. The RNAs were isolated and subjected to RT-PCR of Cdc25A, Cdc25B, and Cdc25C. The previously described primers for real-time PCR were used.

### Statistical Analysis

Statistical analyses were performed using Wilcoxon signed rank tests. Results are shown as mean ± s.d. Significance was inferred for *P*-values of <0.05 (two-tailed).

## Additional Information

**How to cite this article**: Ito, Y. *et al.* Suppression of Poly(rC)-Binding Protein 4 (PCBP4) reduced cisplatin resistance in human maxillary cancer cells. *Sci. Rep.* **5**, 12360; doi: 10.1038/srep12360 (2015).

## Supplementary Material

Supplementary Information

## Figures and Tables

**Table 1:** **The enhancement ratio of mRNA was examined using PCR array**.

**Ratio**	**Genes up-regulated by cisplatin**
2.2501	B-CELL TRANSLOCATION GENE 2 (BTG2)
2.8284	INOSITOL HEXAPHOSPHATE KINASE 3 (IP6K3)
2.2815	POLY(rC)-BINDING PROTEIN 4 (PCBP4)
2.7702	X-RAY REPAIR, COMPLEMENTING DEFECTIVE, IN CHINESE HAMSTER, 3 (XRCC3)

Genes enhanced by cisplatin treatment (1 μg/ml, 6 h) in IMC3CR are listed. The ratio represents fold changes in mRNA expression compared to the control.
