# Supplementary material for: Suppression of Poly(rC)-Binding Protein 4 (PCBP4) reduced cisplatin resistance in human maxillary cancer cells
Source: Sci Rep. 2015 Jul 21;5:12360. doi: 10.1038/srep12360 (PMC4508830; doi:10.1038/srep12360)
Supplement: Supplementary Information [file srep12360-s1.pdf]

Supplementary information

Suppression of Poly(rC)-Binding Protein 4 (PCBP4) reduced cisplatin resistance in human maxillary cancer cells

Yumi Ito[1], Norihiko Narita[1*], Nozomi Nomi[2], Chizuru Sugimoto[1], Tetsuji Takabayashi[1], Takechiyo Yamada[1], Kazuhiro Karaya[3], Hideki Matsumoto[4], and Shigeharu Fujieda[1]

[1]Department of Otorhinolaryngology Head and Neck Surgery, Faculty of Medical Sciences, University of Fukui, 23 Shimoaizuki, Matsuoka, Eiheiji, Fukui, 910-1193, Japan

[2]Department of Otorhinolaryngology, Faculty of Medical Sciences, University of Oita

[3]Division of Bioresearch, Life Science Research Laboratory, Faculty of Medical Sciences, University of Fukui, 23 Shimoaizuki, Matsuoka, Eiheiji, Fukui, 910-1193, Japan

[4]Division of Oncology, Biomedical Imaging Research Center, Faculty of Medical Sciences, University of Fukui, 23 Shimoaizuki, Matsuoka, Eiheiji, Fukui, 910-1193, Japan

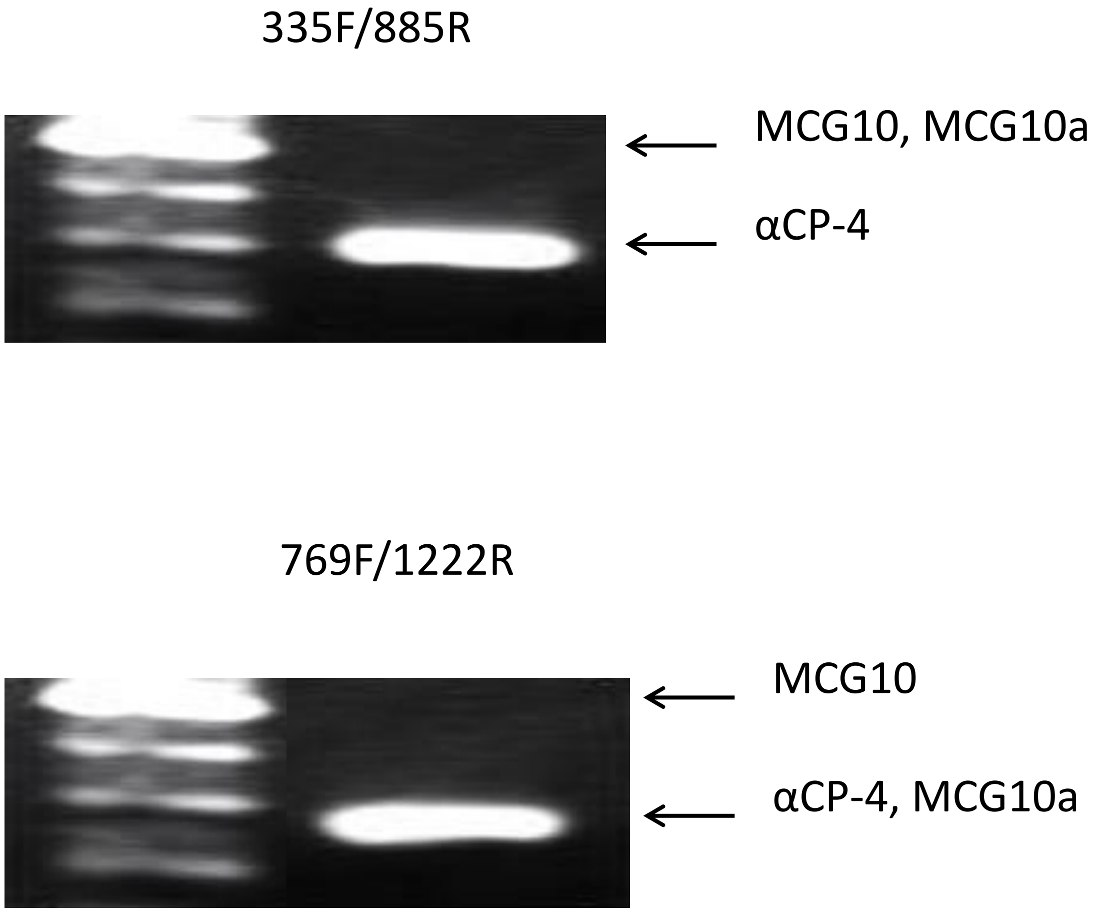

Supplementary Figure S1

The expression of only αCP-4 was confirmed in IMC-3CR cells. The arrows show the positions of expected PCR products of MCG10, MCG10a, and αCP-4.

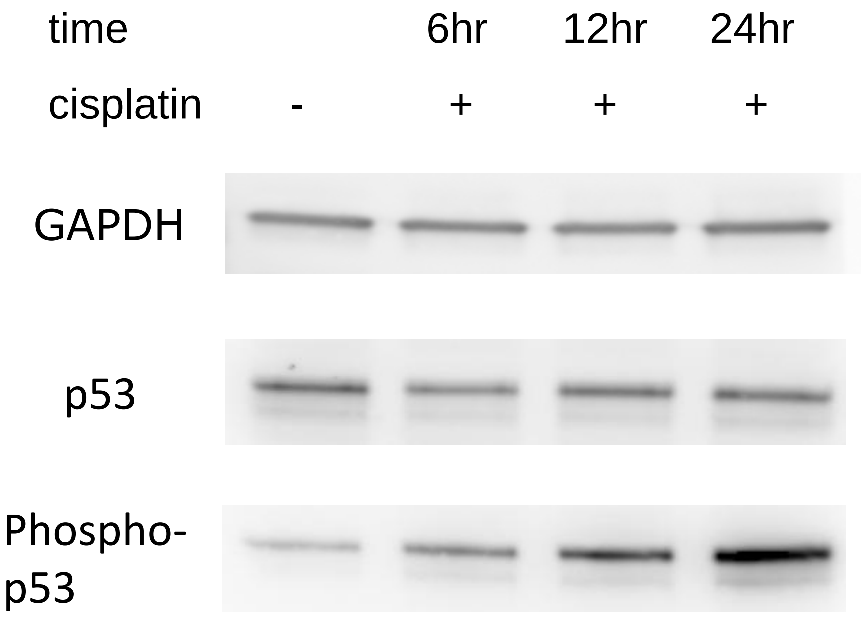

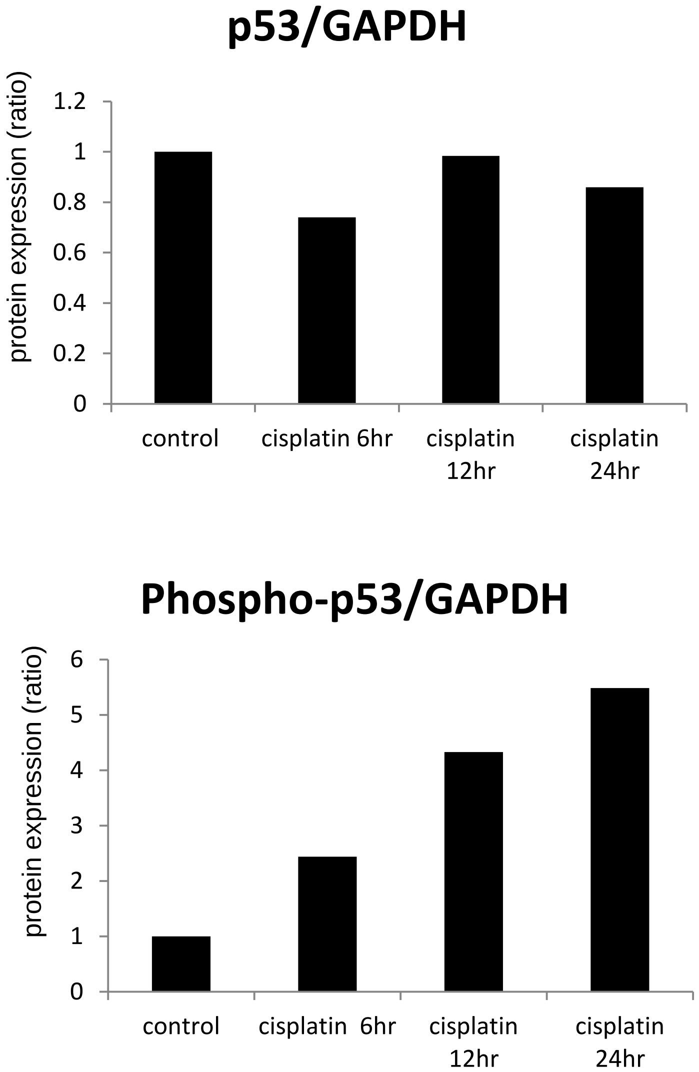

Supplementary Figure S2

Expression of p53 and phosphorylated p53 in IMC-3 CR cells were analyzed by Western blotting (left panel). The density of the Western blotting was quantified (right panel).

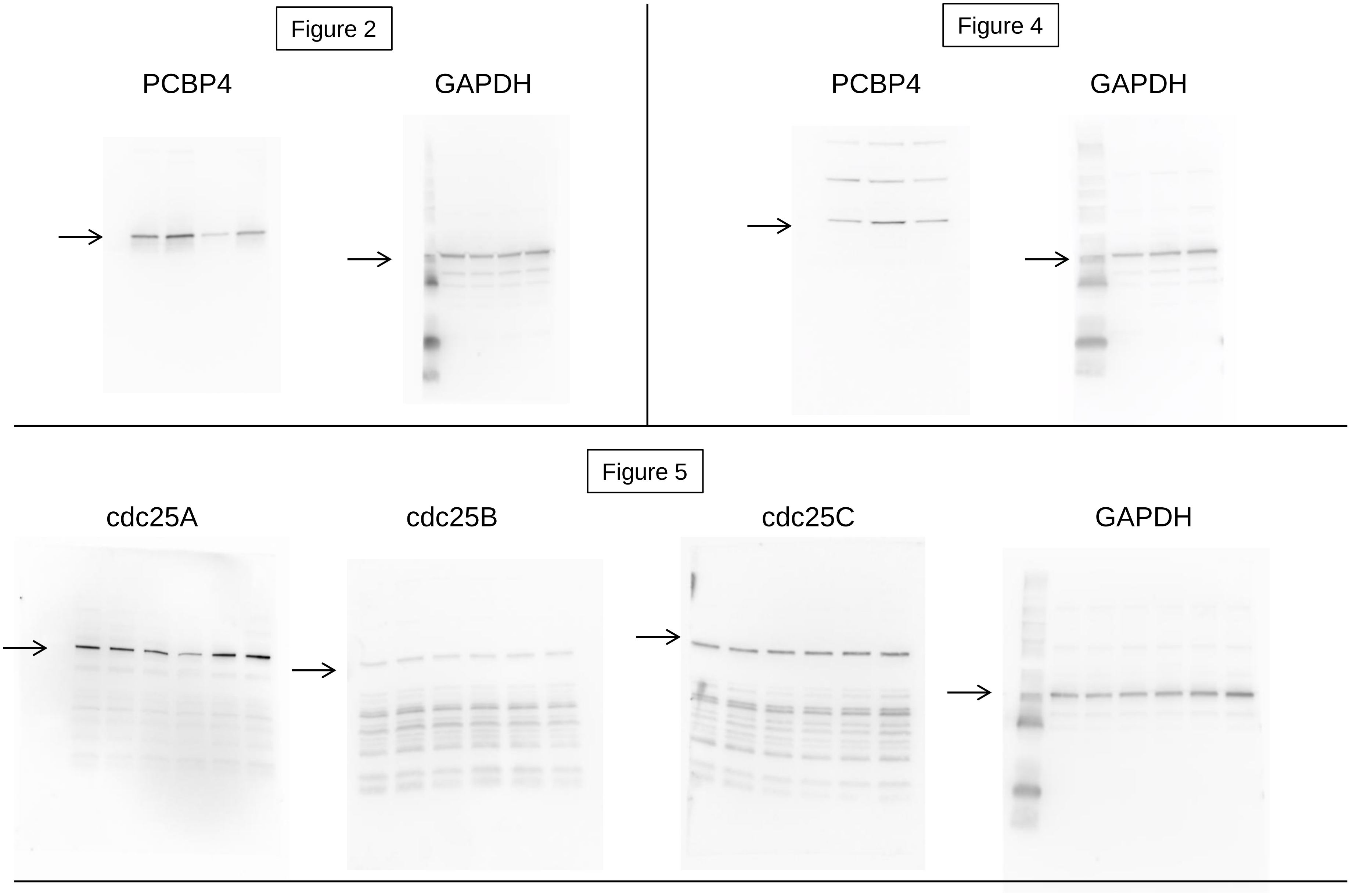

Supplementary Figure S3

Original scans for all of the Western blot. The arrows show the positions of the proteins.

FigureS2

GAPDH

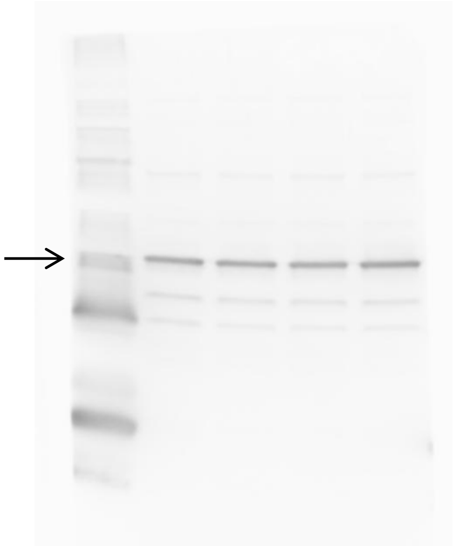

p53

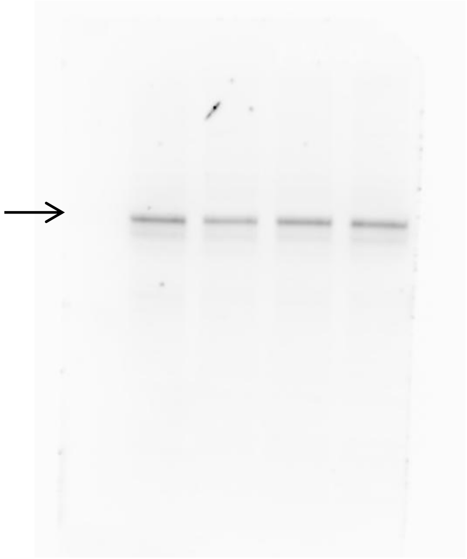

Phospho-p53

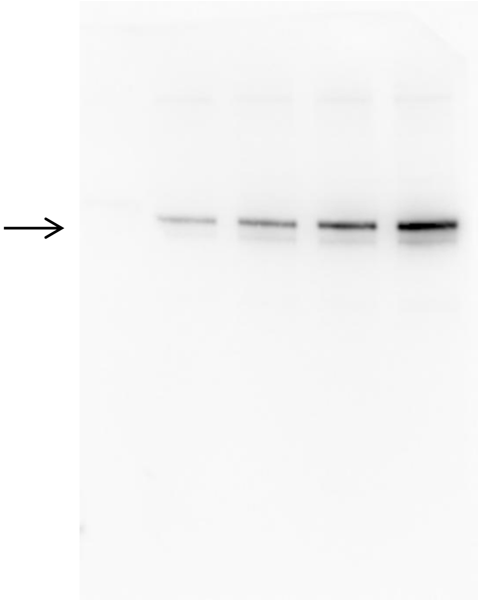

Supplementary Figure S3-2

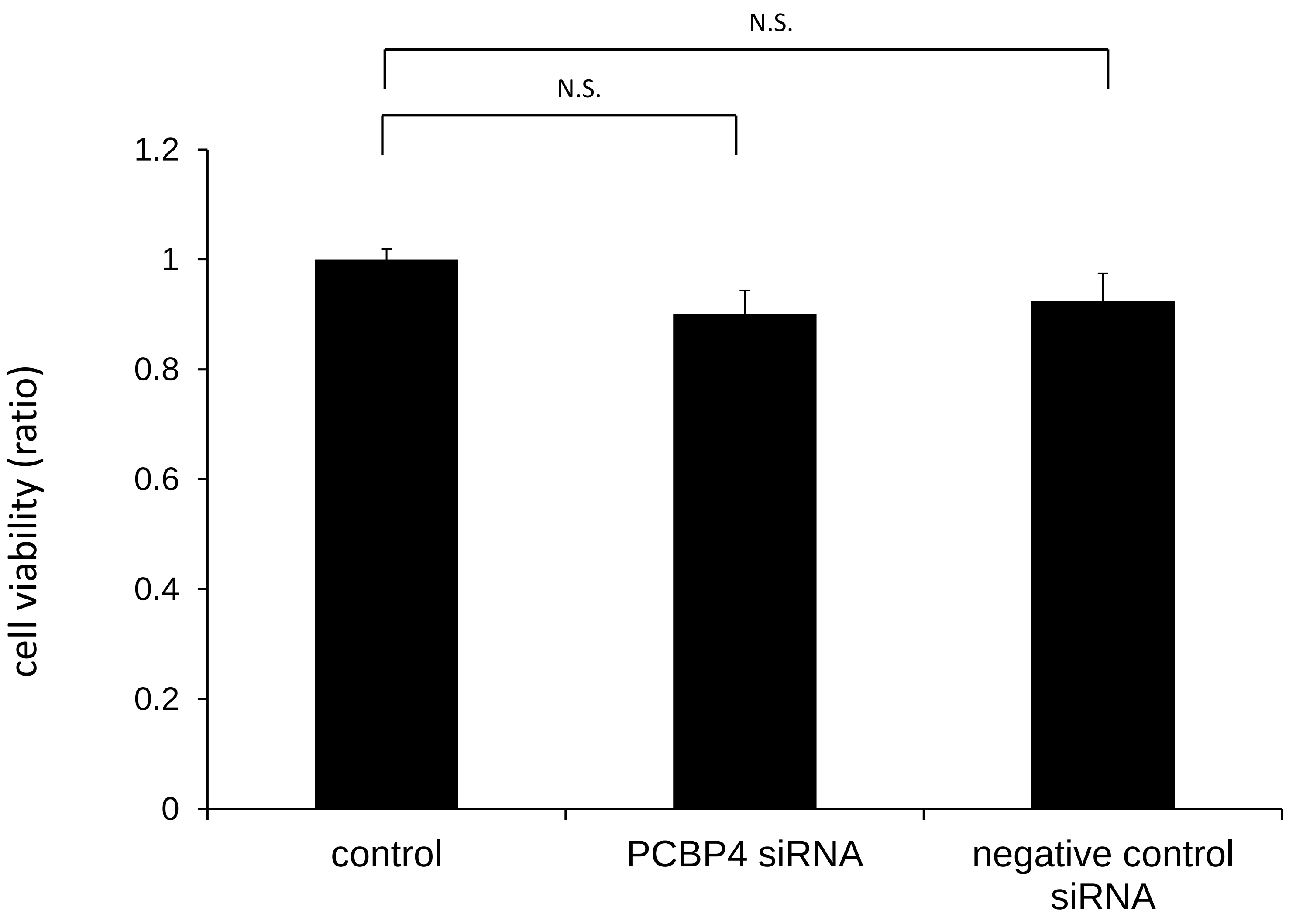

Supplementary Figure S4

Cell viability of IMC-3CR cells 48h after incubation with siRNA for PCBP4 or negative control siRNA. Error bars ± s.d.
